# Retraction: Methylation-mediated repression of microRNA-129-2 suppresses cell aggressiveness by inhibiting high mobility group box 1 in human hepatocellular carcinoma

**DOI:** 10.18632/oncotarget.28694

**Published:** 2025-02-18

**Authors:** Zhikui Liu, Changwei Dou, Bowen Yao, Meng Xu, Linglong Ding, Yufeng Wang, Yuli Jia, Qing Li, Hongyong Zhang, Kangsheng Tu, Tao Song, Qingguang Liu

**Affiliations:** ^1^Department of Hepatobiliary Surgery, The First Affiliated Hospital of Xi’an Jiaotong University, Xi’an, Shaanxi, China; ^*^These authors contributed equally to this work

**This article has been retracted:** Oncotarget has completed its investigation of this paper and determined that many figure images have internal overlaps and duplications. In addition, some of these images were reused in other papers originating from the same lab. As a result, Figures 3B, 3D, 5C, and 5D all contain image duplications and overlaps. In addition, several GAPDH loading control bands in Figure 7, panels A and B, were also used in Figure 8 in a paper published at the same time and from the same lab [1]. Some transwell assay images in Figures 3C and 5D were also reused in a subsequently published paper from that lab [2]. Furthermore, the National Natural Science Foundation of China Supervision Committee has launched an investigation into the suspected academic misconduct of different papers published by Liu Zhikui, Liu Qingguang, Tu Kangsheng, Yang Nan and others from Xi’an Jiaotong University and it was found that this particular paper had problems such as plagiarism, improper manipulation of images, and confusion in the use of images. The investigation demonstrated that the first author, Liu Zhikui, and the corresponding authors were responsible for the problems in the paper. The National Natural Science Foundation of China applied different administrative actions to the above-mentioned authors based on the results of the investigation of their papers published in different journals. In light of these facts, the Editorial decision was made to retract this paper. All authors agreed with the decision.

Original article: Oncotarget. 2016; 7:36909–36923. 36909-36923. https://doi.org/10.18632/oncotarget.9377
